# Relationship Between Occupational Characteristics and Telomere Length in Female Nurses Aged 20–39 Years: A Cross-Sectional Study

**DOI:** 10.3390/healthcare14121657

**Published:** 2026-06-11

**Authors:** Jeonghye Yun, Hyunjung Lee

**Affiliations:** 1 Department of Nursing, Chungnam National University Hospital, 282 Munhwa-ro, Jung-gu, Daejeon 35015, Republic of Korea; 2 College of Nursing, Chungnam National University, 266 Munhwa-ro, Jung-gu, Daejeon 35015, Republic of Korea

**Keywords:** telomere, nurses, work schedule tolerance, burnout, professional, sleep

## Abstract

**Highlights:**

**What are the main findings?**

- Age group was the strongest predictor of telomere length in female nurses aged 20–39 years, with those aged ≥30 showing significantly shorter telomeres than those <30, whereas shift work, burnout, and sleep quality showed no significant associations after controlling for age.
- Despite the lack of a significant link to telomere shortening, a notably high prevalence of burnout (41.2% with high emotional exhaustion) and poor sleep quality (82.4% exceeding the clinical cutoff) was observed among early-career nurses.

**What are the implications of the main findings?**

- The biological manifestations of occupational stress may require longer cumulative exposure to emerge, highlighting the need for longitudinal tracking in older cohorts.
- Saliva-based qPCR demonstrated high measurement reproducibility, supporting its use as a practical, non-invasive method for large-scale occupational health surveillance.

**Abstract:**

**Background:** Korean registered nurses face substantial cumulative occupational stress. Telomere length, a biomarker of cellular aging, is increasingly used in occupational stress research, but evidence on early-career Korean nurses is scarce. This study examined the association between occupational characteristics and telomere length in female nurses aged 20–39 years. **Methods:** Sixty-eight female nurses from a tertiary hospital in South Korea completed the questionnaires. We assessed demographics, occupational factors, burnout (Maslach Burnout Inventory), and sleep quality (Pittsburgh Sleep Quality Index—Korean [PSQI-K]). Salivary telomere length was measured using quantitative polymerase chain reaction (qPCR). Data were analyzed using *t*-tests, ANOVA with a Bonferroni post hoc test, Pearson correlations, and multivariable linear regression. **Results:** Participants showed moderate to high burnout levels (Emotional Exhaustion [EE] = 24.78 ± 10.96), with 41.2% exceeding the high EE threshold. Sleep quality was poor (PSQI-K = 7.90 ± 3.07), with 82.4% exceeding the cut-off. Univariable analyses revealed that younger age, unmarried status, shorter work experience, and higher personal accomplishment were associated with longer telomeres (all *p* < 0.05); multivariable analysis identified only age group as a significant predictor (*B* = −2.055 kb for nurses aged ≥30 years compared to those <30 years, *p* < 0.001). The model explained 83% of the variance in telomere length. Shift work, burnout, and sleep quality were not significantly associated with telomere length after controlling for age. **Conclusions:** Age was the main factor associated with telomere length in young female nurses, suggesting that biological manifestation of occupational effects may require longer exposure. The high prevalence of burnout and sleep disturbances warrants immediate organizational intervention. Saliva-based qPCR demonstrated reliable precision as a non-invasive method for biological monitoring in occupational health research. These findings provide a basis for future longitudinal studies examining the cumulative effects of occupational stress and inform targeted wellness interventions for early-career nurses.

## 1. Introduction

Registered nurses experience distinct occupational characteristics that affect their physical and psychological well-being. These occupational characteristics include prolonged exposure to shift work, chronic sleep deprivation, high levels of emotional and physical stress, heavy workloads requiring constant multitasking, exposure to infectious agents and hazardous substances, and the emotional labor of caring for critically ill patients [1,2,3]. Understanding the biological impact of these occupational factors has become an important research area.

Among these diverse occupational factors, physical hazards such as radiation and chemical exposure are important occupational concerns [4,5]; however, they vary dramatically by unit specialty. For instance, Wilson-Stewart et al. [6] documented that cardiac catheterization nurses receive radiation doses comparable to those of physicians, whereas general ward nurses experience minimal exposure, highlighting the challenge of standardized assessment across diverse nursing specialties. In contrast, this study focused on factors chosen based on their universal prevalence among all registered nurses and their interconnected nature in nursing practice. Specifically, we examined four key characteristics prevalent in the nursing workforce: gender-specific vulnerabilities (as nursing remains a predominantly female profession), shift work patterns, sleep quality, and burnout. In Korea, where 77.6% of hospital nurses work rotating shifts to maintain 24-h patient care, these issues are particularly pronounced [7].

The disruption of circadian rhythms caused by shift work directly contributes to poor sleep quality, which is consistently reported by Korean nurses. Studies have documented widespread sleep disturbances that lead to daytime sleepiness, reduced attention, and increased medical errors [8,9]. This sleep disruption, combined with the demanding nature of nursing work that requires constant collaboration with healthcare professionals while managing heavy workloads and emotional labor, contributes to high burnout rates. Over half of Korean hospital nurses report moderate to severe burnout, with those in tertiary hospitals experiencing additional pressure from complex medical procedures and intense physical and mental demands [10,11].

Against this backdrop, telomere length is a biologically grounded indicator that links cumulative occupational stress to cellular aging. Telomeres, the protective DNA–protein complexes at the ends of chromosomes, progressively shorten with each cell division and serve as biomarkers of biological aging [12]. Chronic stress from occupational factors can accelerate telomere shortening, potentially leading to premature cellular senescence and increased disease risk [13,14]. Various occupational exposures, ranging from chemical hazards in firefighters to psychosocial stress in healthcare workers, are associated with accelerated telomere shortening [15,16,17]. However, research examining the relationship between nursing-specific occupational factors and telomere length has yielded inconsistent results. While some studies reported significant associations between cumulative shift work exposure and telomere shortening [18], others found no clear relationship [19]. Similarly, although international nursing studies have reported associations between burnout and shorter telomeres [16], the evidence remains inconsistent across populations and settings. These inconsistencies may be partly attributed to methodological variations, including differences in the age ranges studied, measurement techniques, and the failure to account for the dominant effect of chronological age on telomere dynamics.

Korean research that systematically examines these relationships remains limited. Recognizing that age is the primary driver of telomere dynamics, we strategically focused on female nurses aged 20–39 years to investigate the biological impact of occupational stress during the formative years of their professional careers [12]. This age-restricted design was employed to control for the dominant, long-term confounding effects of natural age-related telomere attrition observed in older populations [12,20]. Age was analyzed in two groups, the 20s and 30s, a grouping that reflects a distinct career stage in the Korean nursing workforce, where early-career attrition is high and many nurses leave clinical practice within the first few years [7]. To confirm that this categorization did not affect the results, age was also analyzed as a continuous variable (Supplementary Table S1). By narrowing the age range, we aimed to capture the initial biological manifestations of occupational stressors during the critical early-career transition, a period characterized by high physical and emotional demands for Korean nurses. Furthermore, this demographic focus ensures practical relevance given the young age structure of the Korean nursing workforce [7]. Furthermore, we specifically focused on female nurses because evidence indicates that women may experience more pronounced effects of shift-related circadian disruption on sleep quality [21,22]. Accordingly, we investigated the relationships among occupational characteristics, burnout, sleep quality, and telomere length using noninvasive saliva-based testing to provide a baseline for early-career biological monitoring [23]. The specific objectives of this study were as follows:

(1) To assess participants’ general characteristics, occupational factors, burnout, sleep quality, and telomere length.
(2) To analyze telomere length differences according to general and occupational characteristics.
(3) To examine correlations among burnout, sleep quality, and telomere length.
(4) To identify the factors independently associated with telomere length using multivariable analysis.

## 2. Materials and Methods

### 2.1. Study Design

This cross-sectional descriptive study examined the relationships among general characteristics, occupational factors, burnout, sleep quality, and telomere length in female nurses. This study was reported in accordance with the STROBE (Strengthening the Reporting of Observational Studies in Epidemiology) Statement for cross-sectional observational studies (Supplementary Materials).

### 2.2. Participants

The participants were female nurses aged 20–39 years working at Chungnam National University Hospital who understood the purpose of the study and provided informed consent. The exclusion criteria were as follows: (1) acute illness requiring medical treatment within one month, (2) chronic disease medication use, and (3) major personal stressors (bereavement, divorce) within three months.

**Figure 1:**
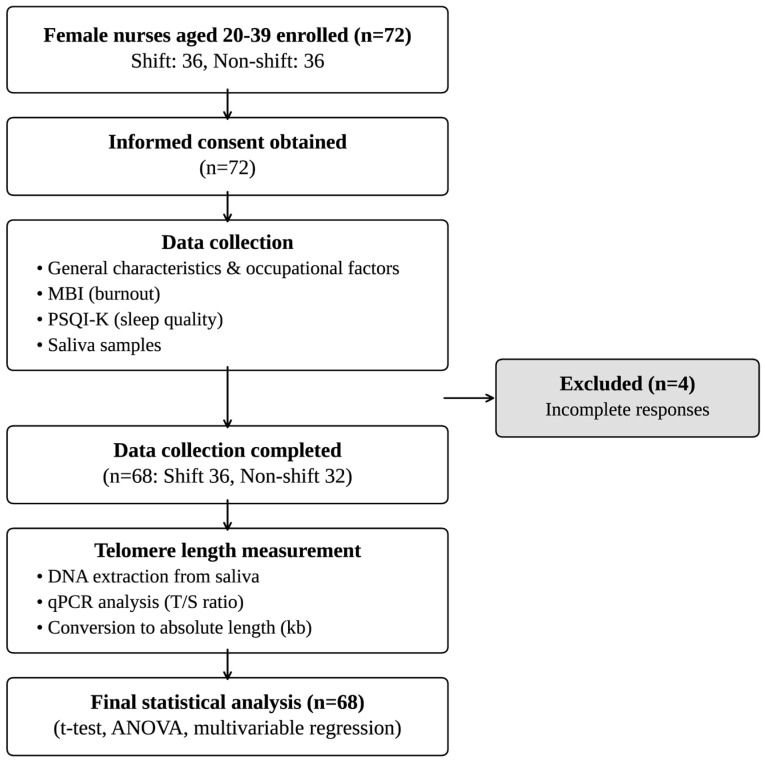
Flow diagram of participant recruitment and study procedures. Of the 72 nurses initially enrolled, 4 were excluded owing to incomplete questionnaire responses (incomplete burnout or sleep assessments, or missing data on other study variables), yielding a final analytic sample of 68 female nurses.

The sample size calculation using G*Power 3.1.9.7 for correlation analysis (α = 0.05, power = 0.80, effect size = 0.30) indicated a minimum of 64 participants. Accounting for 10% attrition, we targeted 72 participants and equally recruited shift and non-shift workers (36 each) to prevent bias. This balanced recruitment strategy was adopted to examine the differential effects of shift work within the nursing population. Non-shift nurses served as an internal reference group, enabling the comparative analysis of shift work impacts among professionals sharing similar educational backgrounds, socioeconomic status, and the general occupational context of hospital nursing. Whereas unit-specific factors (such as patient acuity, workload intensity, and specialty-related stressors) may vary and were not controlled in this study, our focus was on identifying associations between shift work patterns and telomere length within the nursing profession. Of the 72 nurses who provided informed consent, 4 were excluded because of incomplete questionnaire responses, specifically incomplete burnout or sleep assessments or missing data on other study variables. The final analysis therefore included 68 nurses (36 shift workers and 32 non-shift workers) (Figure 1). Although a priori calculation was based on the correlation analysis, a post hoc power analysis confirmed that the achieved sample (n = 68) was well powered for the multivariable regression model (Cohen’s $f^{2}$ = 5.54; statistical power > 99%).

### 2.3. Measurements

#### 2.3.1. Participant Characteristics

General characteristics (age, marital status, and education) and occupational factors (work schedule, years of service, perceived work intensity, job impact on personal life, and turnover intention) were collected using a researcher-developed structured questionnaire comprising 12 items. The specific categories for each variable are listed in Table 1.

#### 2.3.2. Burnout Assessment

Burnout was measured using the Maslach Burnout Inventory (MBI) with permission from the original authors and official purchases from Mind Garden, Inc, Menlo Park, CA, USA [24]. The MBI comprises three subscales, namely Emotional Exhaustion (EE, nine items), Personal Accomplishment (PA, eight items), and Depersonalization (DP, five items), each rated on a seven-point Likert scale ranging from zero (never) to six (daily). Previous Korean nursing studies have confirmed adequate psychometric properties [25]. In this study, Cronbach’s alpha (α) for the MBI was 0.83.

#### 2.3.3. Sleep Quality

Sleep quality was assessed using the Korean version of the Pittsburgh Sleep Quality Index (PSQI-K) [26,27]. This 19-item instrument evaluates seven components: subjective sleep quality, sleep latency, sleep duration, habitual sleep efficiency, sleep disturbances, sleep medication use, and daytime dysfunction. Each component is scored from 0 to 3, yielding total scores of 0–21, with higher scores indicating poorer sleep quality. Scores > 5 indicated clinically significant sleep problems. The Korean version showed a Cronbach’s α of 0.84 [27], while this study’s Cronbach’s α was 0.72.

#### 2.3.4. Telomere Length Measurement

Telomere length was determined by quantitative real-time polymerase chain reaction (qPCR) using DNA extracted from epithelial cells naturally present in whole saliva (the passive drool method). The analysis was conducted in a certified laboratory (Dio-gene, Anyang, Gyeonggi Province, South Korea) using a Bio-Rad CFX96 Real-Time System (Bio-Rad Laboratories, Hercules, CA, USA).

The qPCR assay employed FAM fluorescent dye chemistry to detect telomere amplification, based on established protocols [28,29]. This method quantifies telomere length by comparing the ratio of telomere repeat copy number (T) to a single-copy reference gene number (S) (T/S ratio) in each sample. Standard curves were generated using five-point ten-fold serial dilutions (1, 10, 100, 1000, and 10,000) to ensure optimal PCR efficiency and quantification accuracy. All samples were analyzed in triplicate. Cq (quantification cycle) values were recorded for each reaction. The T/S ratio obtained from the qPCR was then converted to absolute telomere length in kilobases (kb) using a validated conversion algorithm based on the standard curve, allowing for more intuitive interpretation and statistical analysis of telomere length differences between groups.

### 2.4. Data Collection

This study was approved by the Institutional Review Board of Chungnam National University Hospital (IRB No. 2025-02-008) on 14 February 2025. Data were collected between 18 February and 27 February 2025, with the approval of the nursing department. Recruitment notices containing quick response codes were posted on internal bulletin boards. The participants accessed the study information, provided informed consent online, and completed a self-administered questionnaire. Voluntary participation and the right to withdraw from the study at any time were ensured.

Saliva samples were collected using the passive drool method after obtaining written informed consent. The participants were instructed to refrain from eating, smoking, or brushing their teeth for at least 1 h prior to sample collection. All data were anonymized using research codes, stored securely for 3 years following study completion, and destroyed thereafter.

### 2.5. Statistical Analysis

The data were analyzed using IBM SPSS Statistics version 26.0 (IBM Corp., Armonk, NY, USA). Descriptive statistics and normality tests (skewness and kurtosis) were performed. Group differences in telomere length were analyzed using independent *t*-tests or one-way analysis of variance with Bonferroni post hoc tests. Kruskal–Wallis tests were used when the homogeneity of variance was violated. Pearson correlations were used to examine variable relationships.

Multivariable linear regression was used to identify predictors of telomere length. The model included age group and theoretically important occupational variables: shift type, burnout subscales (EE, DP, and PA), and sleep quality (PSQI-K Total). Marital status, education level, and years of service were not included as covariates because they were strongly associated with age group. Model assumptions were verified, including linearity, normality of residuals, homoscedasticity, independence, and multicollinearity. Two sensitivity analyses were conducted: substituting continuous age for the binary age variable (Supplementary Table S1), and additionally adjusting for lifestyle covariates, including body mass index, smoking, alcohol consumption, and exercise frequency (Supplementary Table S2).

## 3. Results

### 3.1. Telomere Length by Participant Characteristics

Table 1 presents the differences in telomere length across participant characteristics. Nurses aged <30 years demonstrated significantly longer telomeres than those ≥30 years (9.46 ± 0.48 vs. 7.37 ± 0.44 kb; *t* = 18.65, *p* < 0.001). Unmarried nurses had longer telomeres than married nurses (8.74 ± 1.09 vs. 7.45 ± 0.67 kb; *t* = −5.51, *p* < 0.001). Bachelor’s degree holders had longer telomeres than those with higher education levels (8.74 ± 1.11 vs. 7.36 ± 0.26 kb; *t* = 5.79, *p* < 0.001).

No significant difference was found between shift and non-shift workers (8.31 ± 1.09 vs. 8.67 ± 1.18 kb; *t* = −1.31, *p* = 0.194). Years of service showed significant differences (*F* = 32.39, *p* < 0.001), with nurses with <6 years of experience displaying longer telomeres than those with ≥6 years of experience.

Although work intensity (*p* = 0.246), job impact on personal life (*p* = 0.303), and turnover intention (*p* = 0.150) were not statistically significant, nurses in the extreme stress categories (‘very high’ intensity, ‘severe impact,’ and ‘planning to change jobs’) numerically exhibited the shortest telomere lengths. However, owing to the small sample size in these extreme subgroups (n < 5), these patterns did not reach statistical significance and should be interpreted with caution.

### 3.2. Burnout, Sleep Quality, and Telomere Length Descriptives

Burnout subscale means were as follows: EE, 24.78 ± 10.96 (skewness = 0.44, kurtosis = 0.40); DP, 7.47 ± 4.61 (skewness = 0.37, kurtosis = −0.73); and PA, 30.07 ± 7.41 (skewness = −0.23, kurtosis = −0.49). The mean PSQI-K total score was 7.90 ± 3.07 (range, 2–16), exceeding the clinical cutoff, with 82.4% (56/68) of participants scoring >5. Mean telomere length was 8.48 ± 1.15 kb (range 6.40–10.00). The qPCR assay showed excellent precision with a technical coefficient of variation (CV) of 0.5% and an internal control CV of 0.7% (Table 2).

### 3.3. Correlations Among Study Variables

Among the burnout subscales, EE and DP showed a positive correlation (*r* = 0.463, *p* < 0.001). EE was significantly correlated with the PSQI-K total score (*r* = 0.448, *p* < 0.001). Telomere length showed a significant positive correlation with PA (*r* = 0.242, *p* = 0.046), indicating that a higher sense of personal accomplishment is associated with longer telomere length. Telomere length showed no significant correlation with EE, DP, or sleep quality (all *p* > 0.05) (Table 3).

### 3.4. Predictors of Telomere Length

Multivariable analysis yielded a significant model (*F* = 56.43, *p* < 0.001) with high explanatory power ($R^{2}$ = 0.85, $adjusted\;R^{2}$ = 0.83). The model assumptions were met, with a tolerance of 0.63–0.96, VIF 1.04–1.58, and Durbin–Watson 1.90. Linearity, normality of residuals (skewness = 0.11), and homoscedasticity were also confirmed. Two sensitivity analyses, using continuous age (Supplementary Table S1) and adjusting for lifestyle covariates (Supplementary Table S2), yielded consistent findings, with age remaining the sole significant predictor (continuous age: *B* = −0.27 per year, *p* < 0.001; lifestyle-adjusted: *B* = −2.07, *p* < 0.001). Among all variables, only age group significantly predicted telomere length, with nurses aged 30 and older exhibiting shorter telomeres than those under 30 (*B* = −2.055, *SE* = 0.120, β = −0.901, *t* = −17.18, *p* < 0.001). Shift type, burnout subscales, and sleep quality were not significant predictors (all *p* > 0.05) (Table 4).

As a sensitivity analysis, an age-only regression model was also fitted, yielding results essentially equivalent to those of the multivariable model (*B* = −2.092, $R^{2}$ = 0.841, $adjusted\;R^{2}$ = 0.838, *p* < 0.001).

## 4. Discussion

In this study, we investigated the relationship between occupational characteristics, burnout, sleep quality, and telomere length in 68 female nurses aged 20–39 years at a tertiary hospital using non-invasive saliva samples. Our findings showed that telomere length was strongly associated with age, whereas occupational factors such as shift work, burnout, and sleep quality showed no significant association after age was taken into account.

Participants exhibited moderate to high burnout levels. Although the mean EE score (24.78 ± 10.96) fell within the moderate range, 41.2% of participants exceeded the high burnout threshold (EE ≥ 27). Notably, the mean PA score (30.07 ± 7.41) fell below the high burnout cutoff (≤31), indicating reduced professional accomplishment across the sample. These findings are consistent with those of a recent meta-analysis indicating a high global prevalence of burnout symptoms among nurses [30].

Sleep quality was notably poor, with mean PSQI-K scores (7.90 ± 3.07) significantly exceeding the clinical cutoff, and 82.4% of participants surpassed this threshold. These findings align with those of large-scale Korean nursing studies that have documented widespread sleep disturbances among nurses [31,32].

Our study demonstrated the reliable precision of saliva-based qPCR for telomere measurement, achieving CV values of 0.5–0.7%. These results support previous research that confirms strong correlations between blood and saliva telomere measurements [23] while offering the advantages of non-invasive collection and reduced participant burden.

Univariable analyses revealed multiple significant associations; however, multivariable analysis identified age as the sole significant predictor of telomere length. The high explanatory power of the model (adjusted *R*^2^ = 0.83) underscores that age is a dominant factor in telomere dynamics. Nurses aged 30 years or older exhibited a 2.055 kb shorter telomere length compared to those under 30 (*p* < 0.001), consistent with established cellular aging patterns [12,20]. However, the restricted age range necessitates cautious interpretation, and subtle occupational effects may require larger sample sizes for detection.

The absence of significant associations between shift work and telomere length aligns with inconsistent findings in previous literature, where the results vary according to exposure duration and intensity [19]. Several explanations are plausible for our young cohort, including insufficient time for cumulative telomere damage manifestation, active telomere repair mechanisms offsetting damage in younger adults [33], and inadequate capture of shiftwork nuances in our classification system [34].

Similarly, overall burnout and sleep quality showed no significant effects on telomere length in the multivariable model, although some studies have reported associations between emotional exhaustion and poor sleep quality with shorter telomere length [16,19,35]. The univariable analysis revealed that a higher sense of personal accomplishment (PA) was significantly associated with longer telomeres (*r* = 0.242, *p* = 0.046), suggesting that personal accomplishment may be associated with telomere maintenance. However, this association did not remain significant after controlling for age, indicating that the PA–telomere relationship may be largely confounded by chronological age. While nurses in the extreme stress categories numerically showed shorter telomeres, these differences were not statistically significant after controlling for the predominant effect of chronological age. This observation warrants further investigation by using alternative analytical methods.

Our findings yield three key implications. First, the predominance of age effects in this young cohort suggests that occupational effects on telomere length may require longer exposure periods or manifest more clearly in older populations. Second, moderate to high levels of burnout and poor sleep quality require immediate organizational attention regardless of their potential association with telomeres. Third, successful implementation of saliva-based testing offers a practical approach for large-scale occupational health monitoring.

These null findings may be better understood in light of the broader literature, in which occupational stress has been linked to shorter telomeres mainly in older workers or after prolonged exposure. For example, work-related exhaustion was linked to shorter telomeres in adults aged 30–64 years [17], and in hospital nurses, shorter telomeres were associated with night-shift work only after about 12 years, with no overall difference between night-shift and non-night-shift workers [18]. Taken together, these findings suggest that the effect of occupational stress on telomere length does not appear right away, but only after exposure builds up over a certain length of time. This points to an exposure-duration threshold: below it, occupational stress is present but cannot yet be detected in telomere length, while above it the effect becomes measurable. Our cohort falls well below this threshold. Our cohort represents a much earlier point: early-career nurses aged 20–39 years with far shorter occupational exposure. Occupational stress was clearly present in this cohort, with 41.2% of nurses exceeding the high-burnout threshold and 82.4% reporting poor sleep quality. Despite this clear exposure, burnout and poor sleep, although highly prevalent, were not reflected in telomere length once age was taken into account. This null result is therefore not in conflict with the earlier studies; on the contrary, it is just what the exposure-duration threshold would predicts at this early-career stage, because the exposure needed for a detectable effect has not yet built up. Rather than contradicting the earlier work, our finding extends it to the lower end of the exposure range and helps show where, over the course of a nursing career, occupational effects on telomere length may begin to appear. To our knowledge, this is the first study to examine telomere length in early-career Korean nurses, and it offers a baseline for future longitudinal studies that track when these effects begin, ideally measuring additional biological markers such as inflammatory markers alongside telomere length.

This study presents both methodological strengths and limitations that should be considered when interpreting the findings. The strengths include the integration of biological markers with psychological assessments, providing complementary biological data alongside self-report measures of burnout and sleep quality. The age-restricted design minimized confounding from age-related telomere attrition, while a balance between the recruited shift and non-shift workers ensured adequate statistical power. Our exclusion criteria helped reduce biological confounders that could obscure occupational effects. The achievement of CV values of 0.5–0.7% using saliva-based qPCR demonstrates that non-invasive methods can provide high measurement reproducibility suitable for occupational health surveillance. The limitations include the cross-sectional design, which might preclude causal inference; the restricted age range, limiting generalizability; and the relatively small sample size, which might have masked subtle occupational effects while potentially inflating the proportion of variance explained by the dominant age variable. The single-center setting also precludes external validity. In addition, inflammatory biomarkers were not measured, lifestyle factors beyond those adjusted for were not assessed, and occupational exposure relied on self-report rather than objective measures. Several further limitations should be noted. First, although the study was well powered to detect the large age effect, the small sample size (n = 68) gave limited power to detect the smaller effects expected for individual occupational factors; the non-significant results for shift work, burnout, and sleep quality may therefore reflect a lack of a detectable effect in this sample rather than evidence that no relationship exists. Second, the cross-sectional design measures telomere length at a single point in time and cannot directly test the exposure-duration threshold described above, which is a question that only longitudinal studies can answer; showing when occupational effects begin to appear will require repeated measurements in the same individuals over time. Third, given these limits, the present study is best understood as a baseline study that establishes reference values and shows that non-invasive, saliva-based telomere monitoring is practical in a previously unstudied population, rather than as a confirmatory test of occupational effects on telomere length. Nevertheless, this study provides a valuable basis for understanding the relationship between occupational stress and biological aging in early-career nurses.

Future research should employ longitudinal designs to track telomere changes over time, expand sample sizes through multi-site collaborations, include broader age ranges to identify age-specific vulnerabilities, and develop more nuanced assessments of shiftwork patterns and intensities. Whereas our study focused on within-profession comparisons, future studies could explore inter-professional comparisons to contextualize nursing-specific risks. Intervention studies testing stress reduction programs, optimized scheduling, and health monitoring systems are necessary to translate our findings into practice.

## 5. Conclusions

In this study, age group was the primary factor associated with telomere length in female nurses aged 20–39 years, with the older group (≥30 years) showing a 2.055 kb shortening compared to the younger group (<30 years). Shift work, burnout, and sleep quality showed no significant associations in the regression analysis; however, the high prevalence of burnout and sleep disturbances requires urgent organizational intervention. Saliva-based qPCR demonstrated consistent reliability in terms of measurement precision within occupational settings, offering a practical alternative to invasive blood sampling. These preliminary findings may serve as a baseline for future longitudinal studies examining when and how cumulative occupational effects on biological aging emerge, and for developing evidence-based interventions to support early-career nurses’ well-being.

## Figures and Tables

**Table 1:** Telomere length by general and professional characteristics (n = 68).

			Telomere Length (kb)		
Variables	Categories	*N* (%)	*M* ± *SD*	*t* or *F*/*H*	*p*-Value
Age	<30	36 (52.9)	9.46 ± 0.48	18.65	<0.001
	≥30	32 (47.1)	7.37 ± 0.44		
Marital Status	Single	54 (79.4)	8.74 ± 1.09	−5.51	<0.001
	Married	14 (20.6)	7.45 ± 0.67		
Education level	Bachelor	55 (80.9)	8.74 ± 1.11	5.79	<0.001
	Master or above	13 (19.1)	7.36 ± 0.26		
Classification	Rotating shift work	36 (52.9)	8.31 ± 1.09	−1.31	0.194
	Fixed day shift	32 (47.1)	8.67 ±1.18		
Years of service	<3 Years ^a^	11 (16.2)	9.41 ± 0.37	^†^ 32.39	^†^ <0.001 ^a,b^
	3 to <6 years ^b^	25 (36.8)	9.24 ± 0.74		>^c,d ‡^
	6 to <9 years ^c^	18 (26.5)	7.75 ± 1.07		
	≥9 years ^d^	14 (20.6)	7.31 ± 0.29		
Intensity of work	Low	3 (4.4)	8.66 ± 1.28	^†^ 4.14	^†^ 0.246
	Moderate	39 (57.4)	8.66 ± 1.16		
	High	23 (33.8)	8.29 ± 1.11		
	Very high	3 (4.4)	7.33 ± 0.23		
Impact of a job on personal life	Mild impact	27 (39.7)	8.65 ± 1.12	^†^ 3.64	^†^ 0.303
	Moderate impact	14 (20.6)	8.48 ± 1.14		
	High impact	24 (35.3)	8.43 ± 1.20		
	Severe impact	3 (4.4)	7.33 ± 0.23		
Intention to change jobs within three months	Never think	20 (29.4)	8.68 ± 1.19	^†^ 5.32	^†^ 0.150
	Occasionally think	35 (51.5)	8.29 ± 1.14		
	Frequently think	9 (13.2)	9.11 ± 0.81		
	Planning to change jobs	4 (5.9)	7.70 ± 1.00		

^†^ Kruskal–Wallis; ^‡^ post hoc = Bonferroni. Groups sharing the same superscript letter do not differ significantly, whereas groups with different letters differ significantly (*p* < 0.05, Bonferroni post hoc test). *M* = mean; *SD* = standard deviation; kb = kilobase.

**Table 2:** Descriptive statistics of burnout, sleep quality, and telomere length (n = 68).

Variables	Categories	*M* ± *SD*	Range (Min–Max)	Skewness	Kurtosis
Burnout (MBI)	EE	24.78 ± 10.96	1–54	0.44	0.40
	DP	7.47 ± 4.61	0–18	0.37	−0.73
	PA	30.07 ± 7.41	13–45	−0.23	−0.49
Sleep quality (PSQI-K)	-	7.90 ± 3.07	2–16	0.82	0.57
Total telomere length (kb)		8.48 ± 1.15	6.4–10.0	−0.07	−1.70

DP = depersonalization; EE = emotional exhaustion; MBI = Maslach Burnout Inventory; PA = personal accomplishment; PSQI-K = Pittsburgh Sleep Quality Index—Korean version; *SD* = standard deviation.

**Table 3:** Correlations among burnout, sleep quality, and telomere length (n = 68).

Variables	EE	DP	PA	PSQI-K Total	Telomere Length
	*r* (*p*)				
EE	1				
DP	0.463 (<0.001)	1			
PA	−0.191 (0.119)	−0.095 (0.440)	1		
PSQI-K Total	0.448 (<0.001)	0.183 (0.136)	−0.140 (0.255)	1	
Telomere length	−0.112 (0.365)	−0.169 (0.168)	0.242 (0.046)	−0.174 (0.157)	1

DP = depersonalization; EE = emotional exhaustion; PA = personal accomplishment; PSQI-K = Pittsburgh Sleep Quality Index—Korean version.

**Table 4:** Factors influencing telomere length: multivariable analysis (n = 68).

Variables	*B*	*SE*	β	*t*	*p*	Tol.	VIF
(Constant)	9.213	0.334		27.54	<0.001		
Age group (ref: <30 years)	−2.055	0.120	−0.901	−17.18	<0.001	0.91	1.10
Shift type (ref: Rotating)	0.100	0.116	0.044	0.86	0.394	0.96	1.04
Burnout subscales (MBI)							
EE	−0.003	0.007	−0.032	−0.51	0.609	0.63	1.58
DP	−0.004	0.014	−0.017	−0.30	0.767	0.77	1.30
PA	0.008	0.008	0.050	0.97	0.338	0.92	1.09
Sleep Quality (PSQI-K Total)	0.009	0.021	0.024	0.42	0.678	0.77	1.30
*R*^2^ = 0.85, Adjusted *R*^2^ = 0.83, *F* = 56.43, *p* < 0.001, Durbin–Watson = 1.90 Max VIF = 1.58							

*B* = unstandardized coefficient; DP = depersonalization; EE = emotional exhaustion; PA = personal accomplishment; *SE* = standard error; Tol. = tolerance; VIF = variance inflation factor; β = standardized coefficient.

## Data Availability

The datasets generated and analyzed during the current study are available from the corresponding author upon reasonable request, subject to ethical approval and participant consent limitations.

## Supplementary Materials

The following supporting information can be downloaded at: https://www.mdpi.com/article/10.3390/healthcare14121657/s1, STROBE checklist; Table S1: Sensitivity analysis with continuous age; Table S2: Sensitivity analysis adjusting for lifestyle covariates. Ref. [36] is cited in Supplementary Materials.

## Abbreviations

The following abbreviations are used in this manuscript:

CV	Coefficient of Variation
DP	Depersonalization
EE	Emotional Exhaustion
MBI	Maslach Burnout Inventory
PA	Personal Accomplishment
PSQI-K	Pittsburgh Sleep Quality Index—Korean version
qPCR	Quantitative Real-Time Polymerase Chain Reaction
T/S ratio	Ratio of the Telomere Signal (T) to the Single-Copy Gene Signal (S)
VIF	Variance Inflation Factors
