# Supplementary material for: Relationship Between Occupational Characteristics and Telomere Length in Female Nurses Aged 20–39 Years: A Cross-Sectional Study
Source: Healthcare (Basel). 2026 Jun 11;14(12):1657. doi: 10.3390/healthcare14121657 (PMC13300521; doi:10.3390/healthcare14121657)
Supplement: Supplementary file 1 [file healthcare-14-01657-s001.zip › healthcare-4269337-supplementary.pdf]

Supplementary Materials

STROBE Statement—Checklist of items that should be included in reports of cross-sectional studies

Relationship between occupational characteristics and telomere length in female nurses aged 20–39 years: A cross-sectional study

Section/Topic	Item #	Recommendation	Reported in
Title and abstract	1	(a) Indicate the study design in the title or abstract.<br>(b) Provide an informative and balanced summary.	Title; Abstract
Background/rationale	2	Explain the scientific background and rationale.	Introduction
Objectives	3	State specific objectives.	Introduction (final paragraph)
Study design	4	Present key elements of study design early in the paper.	Methods 2.1
Setting	5	Describe the setting, locations, and relevant dates.	Methods 2.2, 2.4
Participants	6	(a) Give the eligibility criteria, sources and methods of selection.	Methods 2.2; Figure 1
Variables	7	Clearly define all outcomes, exposures, predictors, potential confounders.	Methods 2.3
Data sources	8	For each variable, give sources of data and details of assessment methods.	Methods 2.3.1–2.3.4
Bias	9	Describe any efforts to address potential sources of bias.	Methods 2.2, 2.5
Study size	10	Explain how the study size was arrived at.	Methods 2.2
Quantitative variables	11	Explain how quantitative variables were handled. Describe groupings and why.	Methods 2.5; Introduction
Statistical methods	12	(a–d) Describe all statistical methods. (e) Describe any sensitivity analyses.	Methods 2.5; Table S1, S2
Participants	13	(a) Report numbers at each stage. (b) Give reasons for non-participation. (c) Flow diagram.	Figure 1
Descriptive data	14	(a) Give characteristics of participants. (b) Indicate missing data.	Results 3.1–3.2; Tables 1–2
Outcome data	15	Report numbers of outcome events or summary measures.	Results 3.2; Table 2
Main results	16	(a) Give unadjusted and adjusted estimates. (b) Report category boundaries.	Results 3.3–3.4; Tables 3–4
Other analyses	17	Report other analyses done, including sensitivity analyses.	Results 3.4; Tables S1–S2
Key results	18	Summarise key results with reference to study objectives.	Discussion
Limitations	19	Discuss limitations of the study.	Discussion
Interpretation	20	Give a cautious overall interpretation of results.	Discussion; Conclusion
Generalisability	21	Discuss the generalisability of the study results.	Discussion

Funding	22	Give the source of funding.	Funding statement

Reference: von Elm E, et al. The STROBE statement: guidelines for reporting observational studies. J Clin Epidemiol. 2008;61(4):344–349.

Table S1. Sensitivity analysis: Multivariable regression with continuous age as predictor (n = 68).

Variables	*B*	*SE*	β	*t*	*P*	Tol.	VIF
(Constant)	15.724	1.139		13.80	<.001		
Age (years)	-0.266	0.032	-0.739	-8.30	<.001	0.86	1.16
Shift type (ref: Rotating)	0.343	0.193	0.150	1.78	.080	0.96	1.04
Burnout subscales (MBI)							
EE	-0.008	0.011	-0.076	-0.73	.470	0.62	1.61
DP	0.002	0.024	0.009	0.10	.921	0.75	1.33
PA	0.006	0.014	0.040	0.46	.645	0.90	1.12
Sleep Quality (PSQI-K Total)	0.019	0.036	0.051	0.52	.602	0.75	1.34
R^2 = .581, Adjusted R^2 = .540, F = 14.10, p < .001, Durbin–Watson = 2.22							

B = unstandardized coefficient; DP = depersonalization; EE = emotional exhaustion; PA = personal accomplishment; SE = standard error; Tol. = tolerance; VIF = variance inflation factor; β = standardized coefficient.

Table S2. Sensitivity analysis: Multivariable regression additionally adjusting for lifestyle covariates (n = 67). Note: One participant with missing BMI data was excluded from this analysis.

Variables	*B*	*SE*	β	*t*	*P*	Tol.	VIF
(Constant)	11.299	0.624		18.11	<.001		
Age group (ref: <30 years)	-2.065	0.125	-0.906	-16.48	<.001	0.87	1.15
Shift type (ref: Rotating)	0.095	0.132	0.042	0.72	.473	0.79	1.26
Burnout subscales (MBI)							
EE	-0.004	0.007	-0.040	-0.61	.545	0.59	1.69
DP	-0.003	0.015	-0.011	-0.17	.863	0.73	1.37
PA	0.010	0.009	0.062	1.14	.261	0.80	1.24
Sleep Quality (PSQI-K Total)	0.012	0.022	0.032	0.53	.595	0.76	1.31
BMI ($kg/m^{2)}$)	-0.001	0.022	-0.003	-0.04	.966	0.88	1.14
Smoking (ref: No)	0.004	0.200	0.001	0.02	.984	0.91	1.10
Alcohol (ref: No)	-0.065	0.169	-0.027	-0.39	.702	0.81	1.23

Exercise (ref: No)	-0.180	0.146	-0.079	-1.23	.224	0.76	1.31
R^2 = .850, Adjusted R^2 = .824, F = 31.85, p < .001, Durbin–Watson = 1.80							

B = unstandardized coefficient; BMI = body mass index; EE = emotional exhaustion; DP = depersonalization; PA = personal accomplishment; PSQI-K = Pittsburgh Sleep Quality Index–Korean version; SE = standard error; Tol. = tolerance; VIF = variance inflation factor; β = standardized coefficient. Smoking was coded as never (reference) vs. ever; alcohol as non-drinker (reference) vs. drinker; exercise as no regular exercise (reference) vs. regular exercise.
